# Lithium intoxication–induced dysgeusia accompanied by glossalgia in a patient receiving lithium carbonate: a case report

**DOI:** 10.1186/s13256-020-02495-6

**Published:** 2020-09-10

**Authors:** Shota Hanyu, Naoko Sugita, Miyuki Matsuda, Toshiya Murai, Hironobu Fujiwara

**Affiliations:** Department of Psychiatry, Kyoto University Hospital, 54 Kawahara-cho, Shogoin, Sakyo-ku, Kyoto-shi, Kyoto, 606-8507 Japan

**Keywords:** Lithium carbonate, Side effect, Dysgeusia, Glossalgia

## Abstract

**Background:**

Lithium carbonate is widely used as a first-line therapeutic agent for the ﻿depressive and manic phases of bipolar disorder. Although limb tremors and hypothyroidism are well-known side effects of lithium carbonate, other rare adverse reactions can also occur.

**Case presentation:**

A 53-year-old Japanese woman diagnosed with lithium intoxication developed dysgeusia and glossalgia during treatment with lithium carbonate. She also showed symptoms of a swaying gait, finger tremors, and dysarthria. All of these symptoms subsided when her blood lithium concentration was reduced to a level below that which induces intoxication.

**Conclusions:**

We present a rare case of lithium carbonate–induced dysgeusia accompanied by glossalgia. Early detection of these symptoms is important in clinical settings because they can be overlooked until patients lose their appetite, which severely impairs their quality of life.

## Introduction

Lithium carbonate (lithium) is a first-line mood stabilizer that reduces the risk of suicide in patients with bipolar disorder. However, attention should be paid to the use of this agent because of its narrow therapeutic concentration range in the blood. Clinicians should monitor serum lithium concentrations as well as endocrine and renal functions [1]. To effectively prevent manic and depressive recurrences of bipolar disorder, lithium should be administered to maintain a blood concentration of 0.60–1.20 mEq/L [2]. Severe side effects develop more often at blood concentrations ≥1.50 mEq/L [3]. Typical side effects include limb tremors, renal dysfunction, and hypothyroidism [1].

Here, we report a case of dysgeusia accompanied by glossalgia as symptoms of lithium intoxication in a patient taking lithium carbonate. This case report highlights the importance of the early detection of these symptoms because they are often unrecognized or overlooked and can cause a loss of appetite that severely impairs the patient’s quality of life. Dysgeusia as a symptom of lithium intoxication is rarely reported. To the best of our knowledge, dysgeusia accompanied by glossalgia has not previously been reported in patients taking lithium carbonate.

## Case presentation

**Fig. 1:**
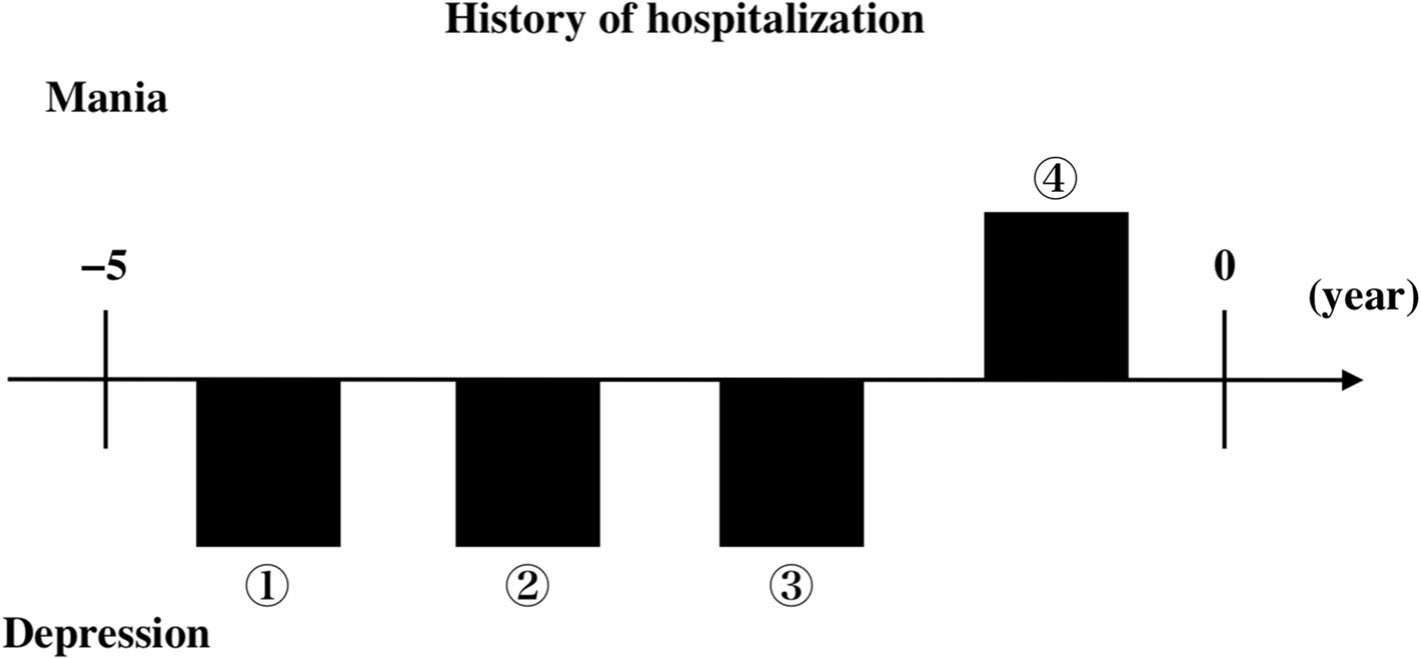
The patient was hospitalized three times for depression and once for mania

Our patient was a 53-year-old Japanese woman. Her sister had bipolar disorder–like episodes (details unknown). When the patient was a high school student, her parents often commented that she was inferior to her sister in appearance. She became further worried about her looks when her teacher told her that she was not suitable to be a tour conductor, which was what she wanted to be. From the age of 18 to 35, she drank approximately 1400 ml of beer every day. However, from the age of 36, she had tried to abstain from alcohol, although she sometimes drank a lot when she felt stressed. Five years before her admission (year − 5), she became unemployed because she was often absent from her job as a result of her mother’s death. Because this caused her to consider herself worthless, she visited a psychiatrist (Fig. 1). She was prescribed antidepressants, including noradrenergic and specific serotonergic antidepressants (NaSSAs) and selective serotonin reuptake inhibitors (SSRIs), to treat her depression. She had ongoing mild suicidal ideation in a state of depression, but she only tried to kill herself once. Most of the time she just stated, “I am meaningless, so I should die,” but did not put this thought into action. Before her current admission (year 0), she had been hospitalized three times for depression and once for mania (Fig. 1). After being discharged from her third hospitalization, she showed a decreased need for sleep and became wasteful with money. It became difficult for her to control her manic state. Thus, she was again admitted to the hospital and was diagnosed with bipolar disorder. She was started on lithium carbonate, and her manic state improved, and she left the hospital. However, she continued to visit the hospital for lingering depression.

One month before her current admission, she had started to experience severe depression, and she subsequently began to lose her appetite as a result of severe dysgeusia and glossalgia. To improve her mental and physical condition, she was admitted to the hospital (day 1). In addition to the aforementioned symptoms, she developed a swaying gait, finger tremors, and dysarthria approximately 1 week before hospitalization. She also vomited and had diarrhea before and after hospitalization. Despite having depression before admission, she was in a manic state upon admission. She stated, “Somehow I am so happy and full of energy.”

Because her swaying gait, finger tremors, and dysarthria had continued for about 1 week, and because she had been prescribed 800 mg/day lithium carbonate (before the current hospitalization, she had received lithium carbonate for approximately 2 years), we investigated cerebellar infarction and lithium intoxication for her differential diagnosis. Her computed tomographic scan showed no intracranial lesions. Blood examinations revealed a blood lithium concentration of 1.99 mEq/L, which is within the range of intoxication. Results of blood tests were mostly within the normal range, except for mean corpuscular volume 104.4 fl, mean corpuscular hemoglobin 34.7 pg, and alkaline phosphatase 458 U/L (Table 1). The patient’s vital signs upon admission included blood pressure of 109/66 mmHg, pulse rate of 84 beats per minute, and body temperature of 36.9 °C. She sometimes complained of pain in her tongue and a taste disorder. Her tongue was diffusely red, but its shape appeared normal on admission. We asked her about dysgeusia and glossalgia, but presumably because of her confused mental state and physically disordered condition as the side effects of lithium treatment, she just said, “I have a pain in my tongue, and I can’t taste anything.” At that time, she needed urgent medical care for her lithium intoxication; therefore, we did not ask her any further questions about her symptoms for the differential diagnosis.

**Table 1:** Laboratory findings on admission

Laboratory test	Value
White blood cells	4220/μl
Red blood cells	360 × 10^4^/μl
Hemoglobin	12.5 g/dl
MCV	104.4 fl
MCH	34.7 pg
Platelets	16.2 × 10^4^/μl
AST	22 U/L
ALT	28 U/L
LDH	196 U/L
Alkaline phosphatase	458 U/L
γ-GTP	20 U/L
Total protein	6.3 g/dl
Albumin	4.1 g/dl
Total bilirubin	0.5 mg/dl
Creatinine	0.71 mg/dl
eGFR	66.7 ml/min/1.73 m^2^
BUN	15 mg/dl
Amylase	67 U/L
Na^+^	138 mEq/L
K^+^	3.7 mEq/L
Cl	109 mEq/L
Mg	1.9 mg/dl
Ca^2+^	9.1 mg/dl
CRP	0.1> mg/dl

Results are from a blood test upon admission

*Abbreviations: ALT* alanine aminotransferase, *AST* aspartate aminotransferase, *BUN* blood urea nitrogen, *Cl* chloride, *CRP* C-reactive protein, *eGFR* estimated glomerular filtration rate, *LDH* lactate dehydrogenase, *MCH* mean corpuscular hemoglobin, *MCV* mean corpuscular volume, *γ-GTP* γ-glutamyl transpeptidase

**Fig. 2:**
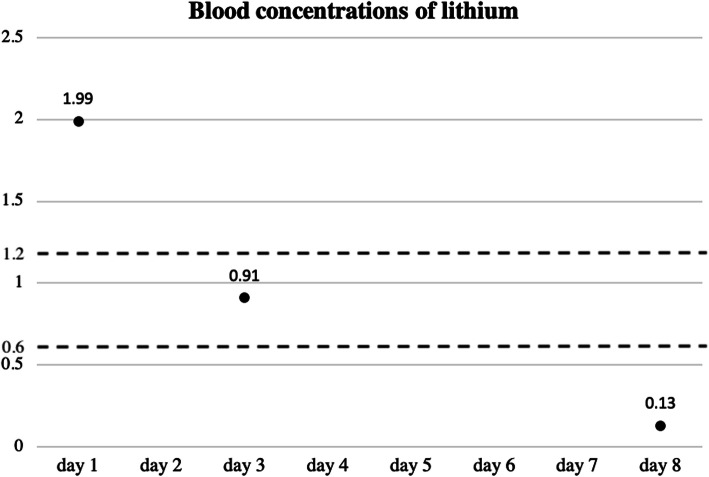
The blood concentration of lithium declined over time. The *dotted lines* indicate the therapeutic range

To treat the patient’s dehydration and increase her urinary excretion of lithium [4], we started infusing physiological saline and stopped prescribing lithium carbonate (day 1). Her blood concentration of lithium declined to 0.91 mEq/L on day 3 and to 0.13 mEq/L on day 8 (Fig. 2). In terms of symptoms, her dysgeusia and glossalgia were relieved on day 4, her dysarthria was relieved on day 5, and her swaying gait and finger tremors were relieved on day 7. By day 8, all of her symptoms had disappeared. Because cessation of lithium carbonate treatment could raise the patient’s risk for the recurrence of bipolar disorder, we continued with follow-up observation of her symptoms of this disorder. During the follow-up observation, she underwent rehabilitation, including occupational therapy, so that she could return to her normal life after being discharged from the hospital. From day 14, her depression reappeared, and an initial dose of 200 mg/day of lithium carbonate was prescribed. Once she had recovered from this depression, she left the hospital (day 30). By the time the patient was discharged, the color of her tongue had returned to normal.

## Discussion

Diagnosis by exclusion and clinical course is essential for the diagnosis of drug-induced disorders. On the basis of information obtained upon admission, the results of a blood sample, and the observed changes in blood lithium concentration, we retrospectively considered several diseases for our patient’s differential diagnosis.

Causes of glossalgia include (1) aphthous stomatitis, (2) oral candidiasis, (3) herpangina, (4) scarlet fever, (5) Hunter’s glossitis, (6) Plummer-Vinson syndrome, (7) Sjögren syndrome, (8) xerostomia, (9) neuralgia, (10) psychogenic pain, (11) drug-induced pain, and (12) idiopathic pain [5]. The shape of our patient’s tongue looked normal, and she did not have a fever, headache, skin rash, or dry mouth; therefore, (1) aphthous stomatitis, (2) oral candidiasis, (3) herpangina, (4) scarlet fever, (7) Sjögren syndrome, and (8) xerostomia can be excluded. Regarding (5) Hunter’s glossitis, it should be treated with cobalamin for 2–4 weeks or folate for 6 months to supplement a deficiency [6, 7]. After admission, our patient ate only hospital meals and did not receive cobalamin or folate preparations. Moreover, her blood vitamin B_12_ concentration on day 3 was normal (295 μg/ml). Although her blood folate concentration was not measured, she rapidly recovered from glossalgia after admission; thus, (5) Hunter’s glossitis is not likely to have been the cause. Regarding (6) Plummer-Vinson syndrome, therapy for this syndrome consists of iron agents for 3 months [8, 9]. However, our patient’s blood test results on day 3 were normal (hemoglobin 12.3 g/dl, iron 126 μg/dl), and it is unlikely that her iron deficiency improved during the first 2 days after admission. Thus, Plummer-Vinson syndrome can also be ruled out. Because the patient’s mental condition was constant for the first 4 hospital days, (10) psychogenic pain is also unlikely to have been the cause. Her glossalgia improved in inverse proportion to her blood lithium concentration; therefore, (11) drug-induced pain is the most likely cause of the glossalgia among (9) neuralgia, (11) drug-induced pain, and (12) idiopathic pain.

Causes of dysgeusia include (a) psychogenic disorders, (b) drug-induced disorders, (c) zinc deficiency, (d) having a cold, (e) systemic diseases, (f) iatrogenic disorder (after oral surgery), (g) iron deficiency, (h) trauma, and (i) idiopathic disorders [10]. Our patient did not have a cold, ongoing systemic disease, or a history of oral surgery or injury. Thus, (a) psychogenic disorders, (d) having a cold, (e) systemic diseases, (f) iatrogenic disorder (after oral surgery), (g) iron deficiency, and (h) trauma were probably not the causes of her dysgeusia. In terms of (c) zinc deficiency, this disorder is treated with zinc supplementation for 5–6 months. After admission, our patient ate only hospital meals and did not receive any zinc supplements [11]. Furthermore, her blood zinc concentration on day 3 was normal (85 μg/dl). Therefore, (c) zinc deficiency can be excluded. Among (b) drug-induced disorders and (h) idiopathic disorders, the most likely cause of the dysgeusia is (b) drug-induced disorders, for the same reason as for the symptom of glossalgia.

These factors therefore suggest that lithium carbonate induced our patient’s dysgeusia and glossalgia.

There are several causes of lithium intoxication. Renal dysfunction can increase the blood concentration of lithium carbonate because it extends its half-life [12]. However, our patient did not have decreased renal function. Other causes of lithium intoxication include overdose, diuretic drugs, and hypovolemia. Before admission, our patient was regularly prescribed 800 mg/day of lithium carbonate. This was her first experience of lithium intoxication, but she had previously overdosed on an antidepressant; therefore, she might potentially have also overdosed on lithium carbonate [13], although she did not state this clearly. She was not receiving diuretic drugs, but she vomited and had diarrhea. Therefore, she might have been dehydrated both before and upon admission.

As shown in Fig. 2, our patient’s blood lithium concentration on day 3 was 0.91 mEq/L, but her dysgeusia and glossalgia symptoms were not relieved until day 4. These results suggest that symptoms can be present even if blood lithium concentrations are within the normal therapeutic range (0.60–1.20 mEq/L). We therefore advise clinicians to pay close attention to these symptoms, regardless of blood lithium concentrations during the course of lithium intoxication treatment, because these side effects may recur. If patients cannot tolerate the side effects of lithium, a switch to valproate, quetiapine, or lamotrigine as a second-line therapy is recommended [14–19]. For patients who do not respond to these drugs or cannot tolerate their side effects, aripiprazole, olanzapine, or risperidone are alternatives. We therefore considered a switch to a different medication as a treatment option for this patient [14, 15, 17, 18]. Currently, however, our patient is leading her life without the recurrence of mania, depression, or adverse drug reactions at a lithium blood concentration of 0.4 mEq/L.

Lithium is considered to affect two intracellular signaling pathways, inositol monophosphate and glycogen synthasekinase-3, which are related to mental stability and neurological function [20]. However, the mechanisms by which lithium causes dysgeusia and glossalgia are unknown. Therefore, further research is needed.

Dysgeusia caused by lithium carbonate treatment is rare. One case report refers to dysgeusia accompanied by dysosmia [21], and other reports mention dysgeusia alone [22–26]. Other case reports on tongue-related side effects of lithium carbonate treatment include geographic tongue, black hairy tongue, and orolingual dyskinesia [27–29]. Therefore, to the best of our knowledge, the present report of lithium-intoxication-induced dysgeusia accompanied by glossalgia has not previously been reported. We believe that these side effects of lithium carbonate occur more frequently than expected. This is partly because patients assume they are unrelated to their medicine or illness and do not mention them to their doctors, and partly because doctors may overlook them or consider them psychogenic. Thus, clinicians should ask patients about the presence of glossalgia and dysgeusia when they prescribe lithium carbonate.

## Conclusion

We present a rare case of lithium carbonate–induced dysgeusia accompanied by glossalgia. This report addresses the importance of its early detection and treatment. Because adverse events rarely occur in the tongue, clinicians often overlook them. In our patient, the discovery of the symptoms was delayed because the patient did not mention them until they became severe and unbearable. This led to a loss of appetite and impaired quality of life. These side effects of lithium carbonate treatment might cause poor medication adherence, which can potentially lead to an exacerbation of the symptoms of bipolar disorder.

## Data Availability

The datasets analyzed in this case report are available from the corresponding author on request.
